# Analysis of Histochemical Characteristics of Submandibular Gland of the Bactrian Camel

**DOI:** 10.3390/vetsci12020108

**Published:** 2025-02-02

**Authors:** Yulu Chen, Guojuan Chen, Yumei Qi, Jianlin Zeng, Long Ma, Xudong Zhang, Xiaolong Qie, Yajuan Jin, Haijun Li, Ligang Yuan

**Affiliations:** 1 College of Veterinary Medicine, Gansu Agricultural University, Lanzhou 730070, China; cylsgxka520@163.com (Y.C.); hopeissnow1990@163.com (G.C.); qym0212@163.com (Y.Q.); zengjl0123@163.com (J.Z.); ml2000416@163.com (L.M.); zxd82598@163.com (X.Z.); qxl19993242837@163.com (X.Q.); jinyj118@163.com (Y.J.); lhj211999@163.com (H.L.); 2 Huangzhong District Animal Disease Prevention and Control Center, Xining 811600, China; 3 Gansu Key Laboratory of Animal Generational Physiology and Reproductive Regulation, Lanzhou 730070, China

**Keywords:** Bactrian camel, submandibular gland, striated tube, EGF, EGFR

## Abstract

The ultrastructure of submandibular gland (SMG) of Bactrian camels was observed by a transmission electron microscope. Routine HE staining, special staining combined with immunohistochemistry, and immunofluorescence techniques were used to study the histochemical characteristics of the submandibular gland and the localisation and distribution characteristics of epidermal growth factor (EGF) and epidermal growth factor receptor (EGFR). HE results showed that the submandibular gland of Bactrian camels was composed of mixed serous and mucinous acini glands. The submandibular striated duct was highly developed and connected with intercalated ducts with larger diameter. Intercalated ducts are shorter and directly connected to acini. In AB-PAS staining, it was observed that the inner wall of striated tube was strongly positive for AB staining. The distribution of the reticular fibres around the follicles and ducts of the submandibular gland is distinct, with collagen fibres distributed mainly in the periphery of the ducts and sparse collagen fibres in the periphery of the acini. Immunohistochemistry and fluorescence show that EGF is strongly positive in striated and intercalated ducts, and EGFR is weakly positive in striated and intercalated ducts. Bactrian camel SMGs secrete more acidic mucins, and EGF and EGFR were mainly secreted and play a role in the pipeline system of SMGs.

## Simple Summary

The histochemical characteristics of the submandibular gland of the Bactrian camel were analysed by the ultrastructural features of the submandibular gland, the expression and distribution of epidermal growth factor, and the epidermal growth factor receptor, which provided a basis for exploring the physiological functions of the submandibular gland of Bactrian camels. This study may provide clues for the analysis of the submandibular gland in Bactrian camels in improving the rumen microenvironment and better adapting to desert forage conditions.

## 1. Introduction

The animal submandibular gland (SMG) is a salivary gland composed of both acini and ducts glands. The acini mainly secrete saliva, which contains inorganic salts, water, mucins, globulins, and salivary amylase, mainly affecting the moistening of the oral cavity, assisting in the digestion of food, defending against microbial infections, and playing an important role in regulating the microenvironment of the animal’s rumen [1]. Otherwise, the ducts are responsible for producing the biologically active substances that regulate physiological activity via blood circulation. More than 30 active peptides have been found to be secreted by the SMG, such as EGF, and EGFR were essential for maintaining the normal function and metabolic activities of the gastric mucosa and intestinal tract [1,2]. EGF is a heat-resistant single-chain low-molecule polypeptide composed of 53 amino acid residues, which are mainly synthesized by SMGs and the duodenum, widely exist in liquids such as breast milk, saliva, urine, intestinal fluids, blood, amniotic fluid, and glands [2]. Studies have shown that EGF is associated with the transport of nutrients such as sodium ions (Na^+^), magnesium ions (Mg^2+^), inorganic phosphorus, glucose, glutamine, and other nutrients, which play an important role in the promotion of animal growth and the improvement of animal performance [3]. EGFR is a kind of multifunctional transmembrane glycoprotein with tyrosine kinase activity and high affinity for EGF and is widely distributed in various epithelial cell membranes [4]. EGF binds to EGFR on target cells and forms a dimer, which activates casein kinase and a series of signalling pathways through self-phosphorylation, induces epithelial cell growth, participates in cell proliferation, differentiation, and angiogenesis, and regulates endocrine secretion and tissue repair, etc. [2].

The SMG is the main source of rumen fluid that helps the ruminants undergo microbial fermentation. EGF and EGFR play important biological activities in the oral and rumen internal environment in ruminants [1]. Our previous studies have shown that the submandibular glands of Tibetan sheep were dominated by serous acinar glands, EGF was mainly distributed in the striated ducts, and the binding site of EGF and EGFR was mainly at the basement membrane of the striated ducts, playing a role in the proliferation of serous acini cells and secretion [5]. Further research found that yak SMGs secreted more acidic mucin and EGF compared to yellow cattle in the same pasture area, which could improve the rumen microenvironment and the decomposition of crude fibre in highland forage [1]. The Bactrian camel is highly adaptable to the desert, with adaptability to cold, drought, saline environments, and the obvious temperature difference between day and night [6]. Bactrian camels have a higher salivary viscosity than other domestic animals, which contributes to enhancing the rumen microenvironment and facilitating the adaptation of the rumen to the unique digestive mechanism in desert environments [7]. The Bactrian camel SMG is a mixed gland. We analysed the histochemical characteristics of the SMG by transmission electron microscopy, special staining, and the expression of EGF and EGFR, which will provide a reference for the research on the physiological functions of the Bactrian camel.

## 2. Materials and Methods

### 2.1. Animals

Two-year-old Bactrian camel (n = 8) SMGs were collected from a designated slaughterhouse in Wuzhong, Ningxia, China. The submandibular gland was removed from the sample in accordance with ethical principles. All experimental animals were approved by the Animal Health and Use Committee of the College of Veterinary Medicine, Gansu Agricultural University (Approval number: GSAU-Eth-VMC-2021-110), and all experiments were conducted in accordance with the relevant guidelines and regulations.

### 2.2. Main Drugs and Reagents

All the experimental antibodies were purchased from commercial suppliers. Rabbit polyclonal antibodies against EGF (Bioss Cat# bs-2008R, RRID:AB_10856305), EGFR (bs-10007R), and an IHC test kit (PV-0023) were purchased from Beijing BIOSS Antibodies, Ltd. (Beijing, China). Fluorescent secondary antibodies (ab150113, Alexa Fluor^®^488; ab150079, Alexa Fluor^®^647) were purchased from Abcam (Cambridge, UK). The DAB colour reagent kit (PA110) was provided by Beijing TIANGEN Biotechnology Ltd. (Beijing, China).

### 2.3. Transmission Electron Microscopy

The SMG tissue samples of Bactrian camels fixed with 2.5% glutaraldehyde were divided into 0.5 × 0.5 × 0.5 cm tablets, (washed with phosphate buffer PBS), fixed with 1% osmic acid for 3 h, and washed with PBS 6 times for 10 min each time. The pieces were dehydrated using graded acetone and then soaked overnight in a mixture of spr epoxy and acetone. The tissues were embedded in epoxy resin and sliced by an ultra-thin microtome into an ultra-thin section of 50 nm. The sections were attached to a copper mesh, stained with 1% uranyl acetate and lead citrate for 20 min, and examined with a JEM-1230 electron microscope (Japan NEC(Please delete ‘Japan NEC’)HT7800, HITACHI, Minato-ku, Tokyo, Japan).

### 2.4. Optical Microscope

The Bactrian camel SMG tissue samples were fixed in 4% paraformaldehyde and rinsed in running water for 24 h before gradient ethanol dehydration (50%, 70%, 80%, 95%, and 100%). Subsequently, samples were made transparent with xylene (50% ethanol: 50% xylene, xylene), embedded using an Epon 812 paraffin embedding machine, and sectioned at a thickness of 4 μm using an ultramicrotome (RM2235, Leica, Weztlar, Germany). Then, the paraffin sections were immersed in xylene, 100%, 90%, 80%, and 70% (*v*/*v*) for 5 min, respectively, and washed with tap water for 5 min. They were stained with HE, PAS staining, AB staining, AB-PAS staining, Modified Masson trichromatic dyeing, and Modified Gordon-Sweets dyeing, respectively, and finally, neutral gum was used for sealing. Images were taken using an Olympus light microscope (DP73, Olympus, Tokyo, Japan).

### 2.5. Immunohistochemistry

Dewaxed sections were boiled in 10 mM sodium citrate buffer (pH 6.0) for antigen extraction. The sections were then blocked with 3% hydrogen peroxide at 37 °C for 15 min. Then, 5% goat serum albumin was blocked at 37 °C for 30 min, and then rabbit antibodies against EGF and EGFR (1:400) were incubated at 4 °C for 12 h. After washing with PBS three times, slices were incubated with biotinised goat anti-rabbit IgG at 37 °C for 15 min. Horseradase-labelled streptavidin solution was added and the cells were washed three times with PBS. DAB was added for 5 min and the nucleus was stained with haematoxylin. The sections were observed under a microscope (DP73, Olympus, Tokyo, Japan).

### 2.6. Double Immunofluorescence [8]

Immunohistochemistry was repeated until the primary antibody was added. Mixed antibody EGF + EGFR was added proportionally and incubated at 4 °C overnight. After washing with PBS, they were incubated with AF488 and AF647 (1:800) at 37 °C for 1 h. The nuclei were stained with DAPI (1:1000). The sections were then rinsed with PBS and sealed with an anti-fluorescence quenching sealant. Finally, images were captured using a fluorescence microscope (RVL-100-G, ECHO, San Ramon, CA, USA).

### 2.7. Statistical Analysis

Six different fields of view (1000×) were randomly selected for each IHC slice, and the distribution density of EGF and EGFR was analysed by semi-quantitative analysis.

## 3. Results

The SMG is encased in a fascial sheath formed by the deep cervical fascia, located between the mandible and the hyoid tongue muscle. Overall, the SMG is a kind of tubular vesicular gland that consists of the acini and the ducts. The anatomical nomenclature of the SMG morphology, its macrophage, plasma cells, collagen fibres, and reticular fibres are in accordance with Nomina Anatomica Veterinaria [9].

### 3.1. Histological Observations of the Submandibular Gland of the Bactrian Camel

**Figure 1:**
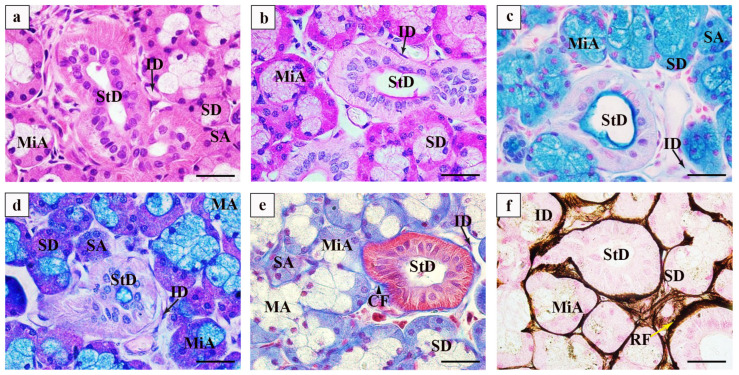
Morphological staining of submandibular glands. (**a**) HE staining of a submandibular gland; (**b**) submandibular gland PAS staining; (**c**) AB staining of a submandibular gland; (**d**) AB-PAS staining of a submandibular gland; (**e**) Modified Masson trichromatic dyeing of a submandibular gland; (**f**) Modified Gordon-Sweets dyeing of a submandibular gland. SA: serous acini; MA: mucinous acini; MiA: mixed acini; StD: striated ducts; ID: intercalated ducts (black arrow); SD: demilune cells; CF: collagen fibres (black triangle); RF: reticular fibres (yellow arrow). Scale bars, 20 μm (**a**–**f**).

We observed the SMG of Bactrian camels using HE staining and found that it is composed of ducts and acini. The vertical longitudinal lines can be seen at the base of the striated tube cells, which consist of simple columnar and cuboidal cells. The intercalated ducts are composed of single, flattened epithelial cells with spindle-shaped or ellipsoid nuclei. The acinus includes mucinous and serous acini. The mucinous acini exhibit light-coloured cytoplasm and rounded nuclei positioned at the base of the vesicle, whereas the serous acini display a darker appearance with oval nuclei, also located at the base of the vesicle. The distal end of the mucinous acini is capped by serous cells, forming a half-moon structure (demilune cells) (Figure 1a). The PAS staining revealed positive purplish-red expressed in the serous acini and demilune cells, with weak positivity observed on the luminal surface of the striated ducts (Figure 1b). The AB staining demonstrated that the positive blue was expressed primarily in the mucous vesicles and on the luminal surface of the striated ducts (Figure 1c). The AB-PAS staining exhibited positive blue expression on the luminal surface of mucous vesicles and striated ducts; however, no significant expression was observed in the demilune cells or intercalated ducts (Figure 1d). The collagen fibres were predominantly distributed surrounding the striated and intercalated ducts (Figure 1e). The reticular fibres were abundantly expressed sparsely around mucinous vesicles (Figure 1f).

### 3.2. Ultrastructural Observation of the Submandibular Gland of the Bactrian Camel

**Figure 2:**
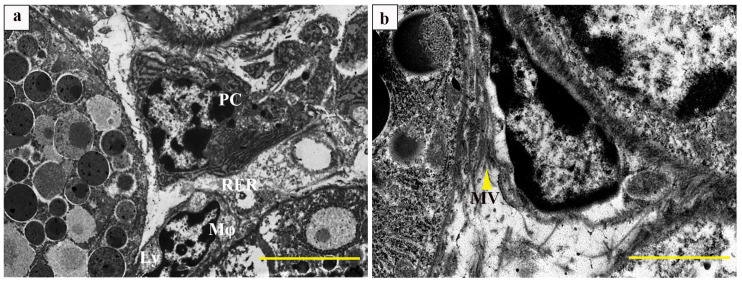
Ultrastructure of the submandibular gland of a Bactrian camel. (**a**,**b**) Ultrastructure of plasma cells and macrophages. Ly: lysosome; PC: plasma cells; Mφ: macrophage; RER: rough endoplasmic reticulum; MV: microvilli (yellow triangle). Scale bars, 5 μm (**a**) and 2 μm (**b**).

Transmission electron microscopy revealed that the plasma cells (PC) contained a substantial amount of rough endoplasmic reticulum, which was tightly arranged in parallel, and the interstitium was filled with amorphous material and contained a small number of lysosomes; the nuclear chromatin was notably abundant and arranged in a whorled pattern. The macrophage (Mφ) nuclei displayed an irregular shape, and the cytoplasm was characterized by the presence of vacuoles and lysosomes (Figure 2a,b).

**Figure 3:**
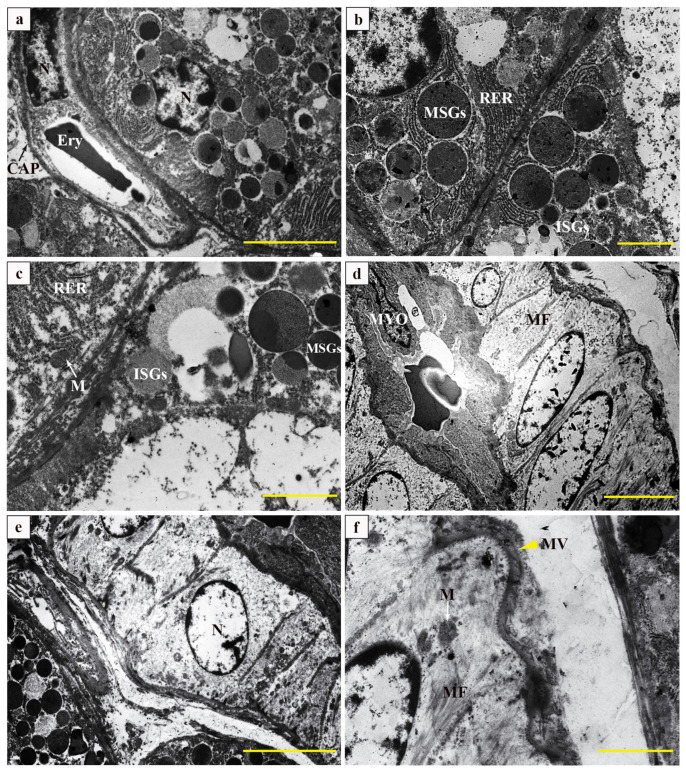
Ultrastructure of the submandibular gland of a Bactrian camel. (**a**–**c**) Ultrastructure of submandibular gland acini of a Bactrian camel; (**d**–**f**) Ultrastructure of striated ducts in the submandibular gland of a Bactrian camel. N: nucleus; CAP: capillary (black arrow); Ery: blood; RER: rough endoplasmic reticulum; MSGs: mature secretory granules; ISGs: immature secretory granules; M: mitochondria (white arrow); MYO: myoepithelial cell; MF: microfilament; MV: microvilli (yellow triangle); scale bars, 5 μm (**a**,**d**,**e**) and 2 μm (**b**,**c**,**f**).

Transmission electron microscopy revealed that serous acini exhibited large, round nuclei containing a substantial amount of rough endoplasmic reticulum and secretory granules in the cytoplasm. The basement membranes of the acini were closely associated with continuous capillaries, characterized by a complex infolding of the plasma membrane and the presence of large vesicle-like structures within the capillary endothelial cells (Figure 3a). The intercellular tubules located at the base of the demilune cells exhibited distinct microvilli, while the serous acinar cells contained numerous mitochondria and an extensive rough endoplasmic reticulum, along with secretory granules of varying sizes, which could be classified as either mature secretory granules (MSGs) or immature secretory granules (ISGs) based on their differing electron densities (Figure 3b). At a magnification of 4000×, the serous acinar cells have a high density of mitochondria and rough endoplasmic reticulum, in addition to a junctional complex that includes tight junctions, intermediate junctions, and desmosomes, facilitating their connection to mucinous cells (Figure 3c). When observed at 1500× magnification, the striatal ducts are distinguished by the vertically arranged mitochondria and infolded plasma membranes at the basal cytoplasm, characterized by small nucleus-to-cytoplasm ratios (Figure 3d). Surrounding the striated duct were small blood vessels (Figure 3e). The epithelium of the striated ducts was interconnected via a junctional complex composed of gap junctions, adherens junctions, and tight junctions; moreover, a significant presence of microvilli was observed at its basal region (Figure 3f).

### 3.3. Comparative Immunohistochemical Analysis of the Bactrian Camel Submandibular Gland

**Figure 4:**
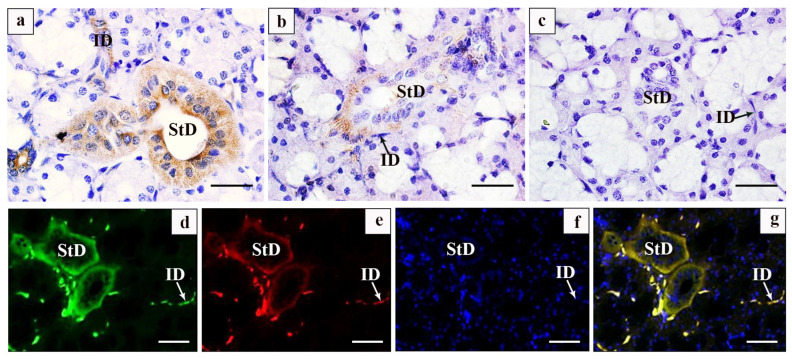
Immunohistochemical distribution characteristics of EGF and EGFR. (**a**) Immunohistochemical expression of EGF in the submandibular gland of a Bactrian camel; (**b**) immunohistochemical expression of EGFR in the submandibular gland of a Bactrian camel; (**c**) Bactrian camel submandibular gland negative control; (**d**) immunofluorescence expression of EGF in a submandibular gland of the Bactrian camel; (**e**) EGFR immunofluorescence expression in the submandibular gland of a Bactrian camel; (**f**) expression of nuclear immunofluorescence in a submandibular gland of a Bactrian camel; (**g**) EGF and EGFR in the submandibular gland of Bactrian camel immunofluorescence double localization. StD: striated ducts; ID: intercalated ducts (white and black arrow). Scale bars, 20 μm (**a**–**c**) and 50 μm (**d**–**g**).

Immunohistochemical analysis revealed a strong positive expression of EGF predominantly in the striated ducts, a positive expression in the intercalated ducts, and an absence of expression in the acini of the SMG of the Bactrian camel (Figure 4a). EGFR had predominant positive expression in both striated and intercalated ducts, with no expression detected in the acini (Figure 4b). There were no expressions in the negative control (Figure 4c). Immunofluorescence staining further confirmed the positive expression of EGF in the striated ducts’ epithelium and a significant expression in the intercalated ducts, while no expression was observed in the acini (Figure 4d). EGFR exhibited weak positive expression in red fluorescence primarily within striated and intercalated ducts, with no detectable positivity in acini or demilune cells (Figure 4e). The nuclei of the cells in the striated and intercalated ducts, as well as in the acini were stained blue (Figure 4f). The dual fluorescence localization revealed that the EGF and EGFR were visualized together in the striated ducts (Figure 4g). Immunofluorescence histochemistry confirmed the absence of EGF and EGFR expression in both serous and mucinous acini in Bactrian camels, as indicated in Table 1.

## 4. Discussion

### 4.1. Histochemical Characteristics of Submandibular Glands of the Bactrian Camel

Salivary glands are abundant with mucin and zymogen granules, primarily synthesising and secreting salivary amylase, which is thinner and clearer in nature and possesses unique digestive and biological functions [10]. The SMG serves as the primary source of saliva in ruminants, consisting of ducts and acini. The ducts consist of intercalated ducts, striated ducts, and excretory ducts, whereas the acini are predominantly mixed acini with a smaller number of serous acini and mucinous acini. Zhou J et al. [11] demonstrated that the number of secretory cells increases with the development of salivary glands by AB and PAS staining techniques. Yang Jie et al. [5] observed that the mucinous acini of SMG from Tibetan sheep and big-tail sheep exhibited positive purplish-red coloration with PAS staining, whereas AB-PAS staining revealed bluish-purple expression in mixed acini along with slightly bluish demilune cells, striated ducts, and intercalated ducts. Furthermore, Yang et al. [1] discovered that yak SMGs secrete more acidic mucous substances to regulate the pH of rumen fluid for enhanced decomposition of the coarse fibres present in forage. The Bactrian camel inhabits desert regions, characterized by significant temperature fluctuations between day and night. Among the three glandular sac regions within their forestomach, those containing glandular lamina propria are classified as mucous glands dominated by acidic-mucin-secreting mucous cells [12], playing a crucial role in host defence mechanisms as well as maintaining a healthy microbial environment [13]. Notably, glycosylation of mucins secreted by serous or mixed acini has been found to be particularly vital for gut microflora [14]. In this study, the SMG of the Bactrian camel predominantly consisted of mixed acini, with serous and mixed acini secreting glycogen and neutral mucin. The mucinous acini and striated ducts primarily contained acidic mucous substances. Semi-quantitative analysis revealed that the area occupied by mucinous acini was larger than that of serous acini, indicating a greater secretion of acidic mucus substances through striated and intercalated ducts into the forestomach during feeding and rumination. This secretion may play a significant role in rumen microbial decomposition and will contribute to the adaptability of the Bactrian camel to harsh environments and their high tolerance for roughage.

The collagen fibrils are the major mechanical component in the extracellular matrix of a broad range of multicellular animals from echinoderms to vertebrates where they provide a stable framework for tissues [15]. The fibrous components of the extracellular matrix can be morphologically categorized into the collagen fibre system and the microfibrillar elastin system [16]. Previous studies have demonstrated that collagen fibres not only enhance tissue and organ stiffness while maintaining structural integrity but also possess growth control and immunomodulatory effects [17]. Reticular fibre primarily consists of type III collagen, which exhibits certain elasticity and widespread distribution throughout the body. Together with collagen fibres, they contribute to the formation of fibrous network scaffolds crucial for maintaining tissue appearance and stiffness [18]. In this study, we observed a predominant distribution of collagen fibres around the intercalated and striated ducts. Additionally, a small amount of collagen fibres were found surrounding acini to preserve acini structure integrity. Reticular fibres were abundant in acini peripheries as well as ducts; even mucinous acini contained some reticular fibres. These findings suggest that the reticular and collagen fibres play a significant supportive role between acini and ducts.

### 4.2. Transmission Electron Microscopy Characteristics of the Submandibular Gland of Bactrian Camels

The function of the conduit system in SMG includes absorbing sodium and discharging potassium, transporting moisture, etc. Redman et al. [19] discovered that the streak formations on the basal of striated ducts cells consist of mitochondria and folded plasma membrane structures, which increases the surface area of the cell base, facilitating the transport of water and electrolytes between the cell and tissue fluid, such as Na+-ATPase and K+-ATPase involved in liquid and ion transport, indicating that striated tubes can modulate saliva acidity–alkalinity through selective reabsorption and electrolyte secretion [20]. It has been demonstrated that the cytoskeletal system, composed of microfilaments in striated ductal wall cells, plays a role in promoting the contraction of ductal cells to enhance salivary secretion [21]. Consistent with this finding, our study reveals that the cytoskeletal system constructed by microfilaments and mitochondria in SMGs was significantly increased in the striated ducts, which played a major role in the reabsorption and transport of saliva. Fletcher et al. [22] discovered that spindle-shaped myoepithelial cells present in the striated ducts possess mechanical stabilization capabilities and contribute to striated duct contraction mechanisms. Our electron microscopic analysis revealed the close adherence between spindle-shaped myoepithelial cells and the luminal surface of striated ducts assisting in the efficient expulsion of saliva. ZHOU et al. [11] proposed that the junction complex between ductal cells composed of desmosomes, intermediate junctions, and tight junctions serves as a fundamental element for glandular function. Lee et al. [23] demonstrated that the perforated capillaries in SMGs contribute to substance permeability and differentiation, and the development of acinar cells. In the present study, the connectivity complex between striated endothelial cells and the capillaries adjacent to the acini, facilitates the drainage of mucinous acini and ion-selective exchange. The antibodies generated by Pc can mediate humoral immunity [24]. The Pc in the salivary gland microenvironment produces autoantibodies and is not dependent on B cell activation and differentiation [25]. Kathrine Skarstein et al. [26] demonstrated that plasma cells from patients with primary dry syndrome actively contribute to inflammation recovery in salivary glands [26]. The Mø, immune cells originating from bone marrow, are distributed across various tissues for phagocytosis and degradation of dead cells, debris, and foreign substances while coordinating inflammatory processes [27]. Mø plays a pivotal role during tissue injury repair by participating in the initiation, maintenance, and regression phases [28]. In this study, the typical structural characteristics of Pc and Mø may significantly contribute to maintaining stability in the Bactrian camel’s SMG microenvironment.

### 4.3. Comparison of EGF and EGFR Expression Analyses in the Submandibular Gland of the Bactrian Camel

The SMG synthesises various cytokines and enzymes, and the epidermal growth factor (EGF) is one of the important bioactive polypeptides. The EGF promotes cell growth and facilitates the proliferation, morphogenesis, and migration of SMG cells [29]. Excision of SMGs in rats significantly reduces the thickness of both gastric mucosa and sinus mucosa [30], while oral administration of EGF leads to a significant increase in gastric wall mucosa thickness in suckling rats, indicating that EGF is an essential trophic factor for the gastric mucosa [31]. EGFR is a receptor for EGF and plays a crucial role in EGF-stimulated growth [32]. The localization of EGFR varies among mammalian salivary glands; however, activation of EGFR induces mucin production [33]. In vitro cell culture studies show that the EGF regulates Ca^+^ concentration and cAMP levels to promote proliferation in rat SMG ductal cells. Additionally, EGFR regulates morphological changes in salivary gland branches by promoting epithelial cell proliferation and maturation while supporting intercellular cell survival [34]. In Tibetan sheep and big-tail sheep SMGs, EGF and EGFR mainly bind in the basement membrane of striate ducts and play a role in the proliferation and secretion of serous acinar cells [5]. In this study, immunofluorescence colocalization experiment analysis revealed the strong positive expression of EGF and EGFR in striated ducts as well as intercalated ducts in Bactrian camel SMG. It is suggested that EGF and EGFR are secreted and combined in striated and intercalated ducts. Since these ducts are responsible for substance transport, they may improve the transport efficiency and secretion of substances. The mechanism of the impact needs to be further investigated.

## 5. Conclusions

In summary, the SMG of the Bactrian camel can secrete higher levels of acid mucin, EGF, and other bioactive substances which contribute to enhancing the rumen microenvironment and facilitating the adaptation of the rumen to the unique digestive mechanism in desert environments. EGF and EGFR are primarily secreted in the ductal system of the SMG, and the mechanisms underlying the improvement of transport efficiency and substance secretion require further investigation.

## Figures and Tables

**Table 1:** Distribution of EGF and EGFR in submandibular gland tissues of Bactrian camels detected by immunofluorescence. -: weak positive expression. ++: medium intensity positive expression. +++: strong positive.

Marker	Expression Site			
	Serous Acinar	Mucous Acinar	Striated Duct	Intercalated Duct
**EGF**	-	-	+++	++
**EGFR**	-	-	++	++

## Data Availability

The datasets used and analysed during the current study are available from the corresponding author upon reasonable request.
